# The big five factors as differential predictors of self-regulation, achievement emotions, coping and health behavior in undergraduate students

**DOI:** 10.1186/s40359-024-01768-9

**Published:** 2024-05-13

**Authors:** Jesús de la Fuente, Paul Sander, Angélica Garzón Umerenkova, Begoña Urien, Mónica Pachón-Basallo, Elkin O Luis

**Affiliations:** 1 University of Navarra, University Campus, Pamplona, 31009 Spain; 2 Faculty of Psychology, University of Almería, Almería, 04001 Spain; 3 Teesside University, Middlesbrough, TS1 3BX UK; 4 Fundación Universitaria Konrad Lorenz, Bogotá, Colombia

**Keywords:** The big five factors, Self-regulation, Achievement emotions and Health Behavior

## Abstract

**Background:**

The aim of this research was to analyze whether the personality factors included in the Big Five model differentially predict the self-regulation and affective states of university students and health.

**Methods:**

A total of 637 students completed validated self-report questionnaires. Using an ex post facto design, we conducted linear regression and structural prediction analyses.

**Results:**

The findings showed that model factors were differential predictors of both self-regulation and affective states. Self-regulation and affective states, in turn, jointly predict emotional performance while learning and even student health. These results allow us to understand, through a holistic predictive model, the differential predictive relationships of all the factors: conscientiousness and extraversion were predictors regulating positive emotionality and health; the openness to experience factor was non-regulating; nonregulating; and agreeableness and neuroticism were dysregulating, hence precursors of negative emotionality and poorer student health.

**Conclusions:**

These results are important because they allow us to infer implications for guidance and psychological health at university.

## Introduction

The personality characteristics of students have proven to be essential explanatory and predictive factors of learning behavior and performance at universities [1–4]. However, our knowledge about such factors does not exhaust further questions, such as which personality factors tend toward the regulation of learning behavior and which do not? Or can personality factors be arranged on a continuum to understand student differences in their emotions when learning? Consequently, the aim of this study was to analyze whether students’ personality traits differentially predict the regulation of behavior and emotionality. These variables align as different motivational-affective profiles of students, through the type of achievement emotions they experience during study, as well as their coping strategies, motivational state, and ultimately health.

### Five-factor model

Previous research has shown the value and consistency of the five-factor model for analyzing students’ personality traits. Pervin, Cervone, and John [5] defined five factors as follows: (1) *Conscientiousness* includes a sense of duty, persistence, and behavior that is self-disciplined and goal-directed. The descriptors *organized, responsible, and efficient* are typically used to describe conscientious persons. (2) *Extraversion* is characterized by the quantity and intensity of interpersonal relationships, as well as sensation seeking. The descriptors *sociable, assertive, and energetic* are typically used to describe extraverted persons. (3) *Openness to experience* incorporates autonomous thinking and willingness to examine unfamiliar ideas and try new things. The descriptors *inquisitive, philosophical, and innovative* are typically used to describe persons open to experience. (4) *Agreeableness* is quantified along a continuum from social antagonism to compassion in one’s quality of interpersonal interactions. The descriptors *inquisitive, kind, considerate, and generous* are often used to describe persons characterized by agreeableness. (5) Finally, neuroticism tends to indicate *negative emotions*. Persons showing neuroticism are often described as moody, nervous, or touchy.

This construct has appeared to consistently predict individual differences between university students. Prior research has documented its essential role in explaining differences in achievement [6, 7], motivational states [8], students’ learning approaches [9], self-regulated learning [10].

### Five-factor model, self-regulation, achievement emotions and health

The relationship between the Big Five factors and self-regulation has been analyzed historically with much interest [11–15]. The dimensions of the five-factor model describe fundamental ways in which people differ from one another [16, 17]. Of the five factors, conscientiousness may be the best reflection of self-regulation capacity. More recent research has shown consistent evidence of the relationship between these two constructs, especially conscientiousness, which has a positive relationship, and neuroticism, which has a negative relationship with self-regulation [18, 19]. The Big Five factors are also related to coping strategies [20].

The evidence on the role of the five-factor model in self-regulation, achievement emotions, and health has been fairly consistent. On the one hand, self-regulation has a confirmed role as a meta-cognitive variable that is present in students’ mental health problems [21]. Similarly, personality factors and types of perfectionism have been associated with mental health in university students [22]. In a complementary fashion, one longitudinal study has shown that personality factors have a persistent effect on self-regulation and health. Sirois and Hirsch [23] confirmed that the Big Five traits affect balance and health behaviors.

### Self-regulation, achievement emotions and health

Self-regulation has recently been considered a significant behavioral meta-ability that regulates other skills in the university environment. It has consistently appeared to be a predictor of achievement emotions [24], coping strategies [25], and health behavior [26]. In the context of university learning, the level of self-regulation is a determining factor in learning approaches, motivation and achievement [27]. Similarly, the self- vs. externally regulated behavior theory [27, 28] assumes that the continuum of self-regulation can be divided into three types: (1) self-regulation behavior, which is the meta-behavior or meta-skill of planning and executing control over one’s behavior; (2) *nonregulation behavior (deregulation)*, where consistent self-regulating behavior is absent; and (3) nonregulation behavior, when regulatory behavior is maladaptive or contrary to what is expected. Some example behaviors are presented below, and these have already been documented (see Table 1). Recently, Beaulieu and collaborators [29] proposed a *self-dysregulation* latent profile for describing subjects with lower scores on subscales regarding extraversion, agreeableness and conscientiousness and higher scores concerning negative emotional facets.

**Table 1:** Conceptual Continuum and Typologies of Each Self-Regulatory Behavior

Characteristics of the person	Self-Regulation (SR)	Non-Regulation or De-regulation (NR)	Dys-Regulation (DR)
Before	Self-analysis of tasks Self-defines goals Self-motivation	No analysis of tasks No goals No motivation	Erroneous self-analysis Erroneous goals Self-demotivation
During	Self-observation Self-analysis Self-correction	No self-observation No supervision No self-correction	Self-distraction Cognitive self-avoidance Self-impediment Procrastination
After	Self-reflection Self-attributions	No reflection No attributions	Erroneous self-assessment Erroneous self-attributions
	**Positive self-affects**	**Mix affects**	**Negative self-affect**

Table 1 here.

Consequently, the question that we pose - as yet unresolved - is whether the different personality factors predict a determined type of regulation on the continuum of regulatory behavior, nonregulatory (deregulatory) behavior and dysregulatory behavior, based on evidence.

### Aims and hypotheses

Based on the existing evidence, the aim of this study was to establish a structural predictive model that would order personality factors along a continuum as predictors of university students’ regulatory behavior. The following hypotheses were proposed for this purpose: (1) personality factors differentially predict students’ regulatory, nonregulatory and dysregulatory behavior during academic learning; they also differentially determine students’ type of emotional states (positive vs. negative affect); (2) the preceding factors differentially predict achievement emotions (positive vs. negative) during learning, coping strategies (problem-focused vs. emotion-focused) and motivational state (engagement vs. burnout); and (3) all these factors ultimately predict student health, either positively or negatively, depending on their regulatory or dysregulatory nature.

## Method

### Participants

Data were gathered from 2019 to 2022, encompassing a total of 626 undergraduate students enrolled in Psychology, Primary Education, and Educational Psychology programs across two Spanish universities. Within this cohort, 85.5% were female, and 14.5% were male, with ages ranging from 19 to 24 years and a mean age of 21.33 years. The student distribution was equal between the two universities, with 324 attending one and 318 attending the other. The study employed an incidental, nonrandomized design. The guidance departments at both universities extended invitations for teacher participation, and teachers, in turn, invited their students to partake voluntarily, ensuring anonymity. Questionnaires were completed online for each academic subject, corresponding to the specific teaching-learning process.

### Instruments

#### Five personality factors

The Big Five Questionnaire [30], based on the version by Barbaranelli et al. [31], assessed scores for five personality factors. Confirmatory factor analysis (CFA) of the 67 scale items resulted in a five-factor structure aligned with the Big Five Model. The outcomes demonstrated satisfactory psychometric properties and acceptable fit indices. The second-order confirmatory model exhibited a good fit (chi-square = 38.273; degrees of freedom (20–15) = 5; *p* > 0.10; chi/df = 7.64; RMR = 0.0425; NFI = 0.939; RFI = 0.917; IFI = 0.947; TLI = 0.937; CFI = 0.946; RMSEA = 0.065; HoeLength index = 2453 (*p* < 0.05) and 617 (*p* < 0.01)). Internal consistency of the total scale was also strong (alpha = 0.956; Part 1 = 0.932 and Part 2 = 0.832; Spearman-Brown = 0.962 and Guttman = 0.932).

*Self-Regulation*: The Short Self-Regulation Questionnaire (SSRQ) [32] gauged self-regulation. The Spanish adaptation, previously validated in Spanish samples [33], encompassed four factors measured by a total of 17 items. Confirmatory factor analysis confirmed a consistent factor structure (chi-square = 845.593; df = 113; chi/df = 7.483; RMSM = 0.0299; CFI = 0.959, GFI = 0.94, AGFI = 0.96, RMSEA = 0.059). Validity and reliability values (Cronbach’s alpha) were deemed acceptable (total (α = 0.86; Omega = 0.843); goal-setting planning (α = 0.79; Omega = 0.784); perseverance (α = 0.78; Omega = 0.779); decision-making (α = 0.72; Omega = 0.718); and learning from mistakes (α = 0.72; Omega = 0.722)), comparable to those of the English version. Example statements include: “I usually keep track of my progress toward my goals,” “In regard to deciding about a change, I feel overwhelmed by the choice,” and “I learn from my mistakes.”

#### Positive-negative affect

The Positive and Negative Affect Scale (PANAS-N) [34], validated with university students, assessed positive and negative affect. The PANAS comprises two factors and 20 items, demonstrating a consistent confirmatory factor structure (chi-square = 1111.147; df = 169; chi/df = 6.518; RMSM = 0.0346; CFI = 0.955, GFI = 0.963, AGFI = 0.96, RMSEA = 0.058). Validity and reliability values (Cronbach’s alpha) were acceptable (total (α = 0.891; Omega = 0.857); positive affect (α = 0.8199; Omega = 0.784); and negative affect (α = 0.795; Omega = 0.776), comparable to those of the English version. Sample items include “I am a lively person, I usually get excited; I have bad moods (I get upset or irritated).”

*Learning Achievement Emotion*: The variable was measured using the Spanish version [35] of the Achievement Emotions Questionnaire (AEQ-Learning) [36], encompassing nine emotions (enjoyment, hope, pride, relief, anger, anxiety, hopelessness, shame, and boredom). Emotions were classified based on valence (positive or negative) and activation (activating or deactivating), resulting in four quadrants. Another classification considered the source or trigger: the ongoing activity, prospective outcome, or retrospective outcome. Psychometric properties were adequate, and the confirmatory model displayed a good fit (chi-square = 529.890; degrees of freedom = 79; chi/df = 6.70; SRMR = 0.053; *p* > 0.08; NFI = 0.964; RFI = 0.957; IFI = 0.973; TLI = 0.978, CFI = 0.971; RMSEA = 0.080; HOELTER = 165 (*p* < 0.05) and 178 (*p* < 0.01)). Good internal consistency was found for the total scale (Alpha = 0.939; Part 1 = 0.880, Part 2 = 0.864; Spearman-Brown = 0.913 and 884; Guttman = 0.903). Example items include Item 90: “I am angry when I have to study”; Item 113: “My sense of confidence motivates me”; and Item 144: “I am proud of myself”.

*Engagement-Burnout*: Engagement was assessed using a validated Spanish version of the Utrecht Work Engagement Scale for Students [37], demonstrating satisfactory psychometric properties for Spanish students. The model displayed good fit indices, with a second-order structure comprising three factors: vigor, dedication, and absorption. Scale unidimensionality and metric invariance were verified in the samples assessed (chi-square = 592.526, *p* > 0.09; df = 84, chi/df = 7.05; SRMR = 0.034; TLI = 0.976, IFI = 0.954, and CFI = 0.923; RMSEA = 0.083; HOELTER = 153, *p* < 0.05; 170 *p* < 0.01). Cronbach’s alpha for this sample was 0.900 (14 items); the two parts of the scale produced values of 0.856 (7 items) and 0.786 (7 items).

*Burnout*: The Maslach Burnout Inventory (MBI) [38], in its validated Spanish version, was employed to assess burnout. This version exhibited adequate psychometric properties for Spanish students. Good fit indices were obtained, with a second-order structure comprising three factors: exhaustion or depletion, cynicism, and lack of effectiveness. Scale unidimensionality and metric invariance were confirmed in the samples assessed (chi-square = 567.885, *p* > 0.010, df = 87, chi/df = 6.52; SRMR = 0.054; CFI = 0.956, IFI = 0.951, TLI = 0.951; RMSEA = 0.071; HOELTER = 224, *p* < 0.05; 246 *p* < 0.01). Cronbach’s alpha for this sample was 0.874 (15 items); the two parts of the scale were 0.853 (8 items) and 0.793 (7 items).

*Strategies for coping with academic stress*: The Coping Strategies Scale (Escala Estrategias de Coping - EEC) [39] was utilized in its original version. Constructed based on the Lazarus and Folkman questionnaire [40] using theoretical-rational criteria, the original 90-item instrument resulted in a 64-item first-order structure. The second-order structure comprised 10 factors and two significant dimensions. A satisfactory fit was observed in the second-order structure (chi-square = 478.750; degrees of freedom = 73, *p* > 0.09; chi/df = 6.55; RMSR = 0.052; NFI = 0.901; RFI = 0.945; IFI = 0.903, TLI = 0.951, CFI = 0.903). Reliability was confirmed with Cronbach’s alpha values of 0.93 (complete scale), 0.93 (first half), and 0.90 (second half); Spearman-Brown coefficient of 0.84; and Guttman coefficient of 0.80. Two dimensions and 11 factors were identified: (1) Dimension: emotion-focused coping—F1. Fantasy distraction; F6. Help for action; F8. Preparing for the worst; F9. Venting and emotional isolation; F11. Resigned acceptance. (2) Dimension: problem-focused coping—F2. Help seeking and family counsel; F10. Self-instructions; F10. Positive reappraisal and firmness; F12. Communicating feelings and social support; F13. Seeking alternative reinforcement.

*Student Health Behavior*: The Physical and Psychosocial Health Inventory [41] measured this variable, summarizing the World Health Organization (WHO) definition of health: “Health is a state of complete physical, mental, and social well-being and not merely the absence of disease or infirmity.” The inventory focused on the impact of studies, with questions such as “I feel anxious about my studies.” Students responded on a Likert scale from 1 (strongly disagree) to 5 (strongly agree). In the Spanish sample, the model displayed good fit indices (CFI = 0.95, GFI = 0.96, NFI = 0.94; RMSEA = 0.064), with a Cronbach’s alpha of 0.82.

### Procedure

All participants provided informed consent before engaging in the study. The completion of scales was voluntary and conducted through an online platform. Over two academic years, students reported on five distinct teaching-learning processes, each corresponding to a different university subject they were enrolled in during this period. Students took their time to answer the questionnaires gradually throughout the academic year. The assessment for Presage variables took place in September-October of 2018 and 2019, Process variables were assessed in the subsequent February-March, and Product variables were evaluated in May-June. The procedural steps were ethically approved by the Ethics Committee under reference 2018.170, within the broader context of an R&D Project spanning 2018 to 2021.

### Data analysis

The ex post facto design [42] of this cross-sectional study involved bivariate association analyses, multiple regression, and structural predictions (SEMs). Preliminary analyses were executed to ensure the appropriateness of the parameters used in the analyses, including tests for normality (Kolmogorov-Smirnov), skewness, and kurtosis (+-0.05).

#### Multiple regression

Hypothesis 1 was evaluated using multiple regression analysis through SPSS (v. 26).

#### Confirmatory factor analysis

To test Hypotheses 2 and 3, a structural equation model (SEM) was employed in this sample. Model fit was assessed by examining the chi-square to degrees of freedom ratio, along with RMSEA (root mean square error of approximation), NFI (normed fit index), CFI (comparative fit index), GFI (goodness-of-fit index), and AGFI (adjusted goodness-of-fit index) [43]. Ideally, all these values should surpass 0.90. The adequacy of the sample size was confirmed using the Hoelter index [44]. These analyses were conducted using AMOS (v.22).

## Results

### Prediction results

The predictive relationships exhibited a continuum along two extremes. On the one hand, conscientiousness, extraversion and openness were significant, graded, and positive predictors of self-regulation. On the other hand, Agreeableness and Neuroticism were negative, graded predictors of self-regulation. A considerable percentage of explained variance was observed (*r*^2^ = 0.499). The most meaningful finding, however, is that this predictive differential grading is maintained for the rest of the variables analyzed: positive affect (*r*^2^ = 0.571) and negative affect (*r*^2^ = 0.524), achievement emotions during study, engagement burnout, problem- and emotion-focused coping strategies, and student health. See Table 2.

**Table 2:** Predictions between the *Five Factor Model* (FFM) and health variables (*n* = 637)

DV	C	E	O	A	*N*	Df	F	Eta Sq.
SR	0.594**	0.107**	0.080*	− 0.171**	− 0.267**	5,488	97,243**	0.499
Pos.A	0.306**	0.377**	0.096*	0.159**	− 0.070*	5,746	100,148**	0.571
Neg.A	0.040	− 0.327**	0.064	0.146**	0.636**	5,736	82,572**	0.524
Pe.S	0.425**	0.267**	0.108	− 0.039	− 0.022	5,430	57,726**	0.401
Ne.S	− 0.259**	− 0.243**	− 0.112**	0.115*	0.426**	5,538	46,271**	0.380
ENG	0.420**	0.224**	0.055	− 0.025	− 0.084*	5,485	52,332**	0.350
BURN	− 0.291**	− 0.061	-0.198**	0.038	0.258**	5,485	39,392**	0.292
PFCS	0.332**	0.407**	0.248**	0.386**	− 0.098	5,269	16,363**	0.233
EFCS	− 0.145**	0.002	− 0.074	− 0.043	0.379**	5,265	13,085**	0.198
**HEALTH**	0.259**	0.251**	− 0.013	− 0.060	− 0.469**	5,134	19,857**	0.426

Note. DV = Dependent Variable; C = Conscientiousness; E = Extraversion; O = Openness to experience; A = Agreeableness; N = Neuroticism; SR = Self-Regulation; Pos.A = Positive Affect; Neg.A = Negative Affect; Pe.S = Positive emotions during study; Ne.S = Negative emotions during study; ENG = Engagement; BURN = Burnout; EFCS = Emotion-focused coping strategies; PFCS = Problem-focused coping strategies:. * *p <* 0.01; ** *p <* 0.001

### Structural prediction results

#### Structural prediction model

Three models were tested. *Model 1* proposes the exclusive prediction of personality factors on the rest of the factors, not including self-regulation. *Model 2* evaluated the predictive potential of self-regulation on the factors of the Big Five model. *Model 3* tested the ability of the Big Five personality traits to predict self-regulation and the other factors. The latter model presented adequate statistical values. These models are shown in Table 3.

**Table 3:** Models of structural linear results of the variables

	Chi^2^	df	CH/df	SRMR	*p*>	NFI	RFI	IFI	TLI	CFI	RMSEA	HOELT	
**1**	417,690	36	11.602	0.10	0.06	0.859	0.894	0.869	0.813	0.868	0.082	0.192	0.220
**2**	320,685	34	9.432	0.08	0.08	0.879	0.765	0.890	0.784	0.889	0.073	0.238	0.274
**3**	455,413	65	7.006	0.04	0.10	0.957	0.951	0.961	0.959	0.961	0.060	0.309	0.344

#### Models of the linear structural results of the variables

##### Direct effects

The statistical effects showed a direct, significant, positive predictive effect of the personality factors C (Conscientiousness) and E (Extraversion) on self-regulation. The result for factor O (openness to experience) was not significant. Factors A (agreeableness) and N (neuroticism) were negatively related, especially the latter. In a complementary fashion, factors C and E showed significant, positive predictions of positive affect, while O and A had less strength. Factor N most strongly predicted *negative affect.*

Moreover, self-regulation positively predicted positive achievement emotions during study and negatively predicted negative achievement emotions. Positive affect predicted positive emotions during study, engagement, and problem-focused coping strategies; negative affect predicted negative emotions during study, burnout, and emotion-focused strategies. Positive emotions during study negatively predict negative emotions and burnout. Engagement positively predicted problem-focused coping and negatively predicted burnout. Finally, problem-focused coping also predicted emotion-focused coping. Emotion-focused coping negatively predicts health and well-being.

##### Indirect effects

**Fig. 1:**
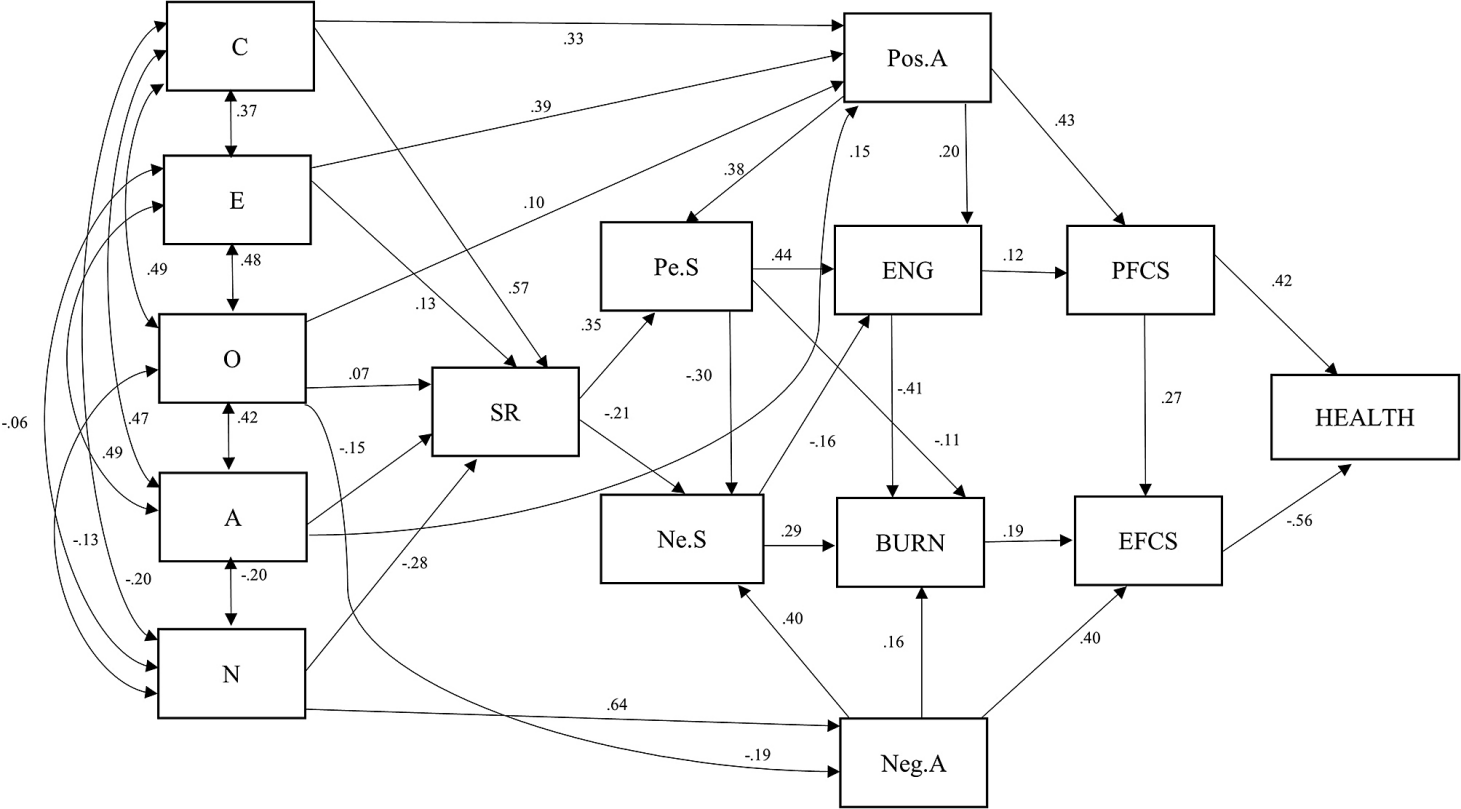
Figure 1

The Big Five factors exhibited consistent directionality. Factors C and E positively predicted positive emotions, engagement, problem-focused coping, and health and negatively predicted negative emotions and burnout. Factor O had low prediction values in both negative and positive cases. Factors A and N were positive predictors of negative emotions during study, burnout, emotion-focused coping and health, while the opposite was true for factors C and E. These factors had positive predictive effects on self-regulation, positive affect, positive emotions during study, engagement, problem-focused strategies and health; in contrast, the other factors had negative effects on negative affect, negative emotions during study, burnout, emotion-focused strategies and health. See Table 4; Fig. 1.

**Table 4:** Total, indirect, and direct effects of the variables in this study, and 95% bootstrap confidence intervals (CI)

*P*.V	Criterion Variable	Direct Effect	Indirect Effect	Total Effect	Results Effects	CI (95%)
C->	SR	0.572		0.572	D.O	(0.42, 0.64)
E->	SR	0.127		0.127	D.O	(0.01, 0.25)
O->	SR	0.074		0.074	D.O	(-,03, 0.14)
A->	SR	− 0.148		− 0.148	D.O	(-0.02, 0.34)
N->	SR	− 0.280		− 0.280	D.O	(-,36, − 0.19)
C->	Pos.A	0.330		0.330	D.O	(0.26, 0.44)
E->	Pos.A	0.339		0.339	D.O	(0.25, 0.41)
O->	Pos.A	0.103		0.103	D.O	(0.02, 0.21)
A->	Pos.A	0.153		0.153	D.O	(0.01, 0.32)
N->	Pos.A				N.E	
C->	Neg.A	0.192		0.192	D.O	(0.02, 0.25)
E->	Neg.A				N.E	
O->	Neg.A				N.E	
A->	Neg.A				N.E	
N->	Neg.A	0.673		0.673	D.O	(0.47, 0.74)
C->	Pe.S		0.329	0.329	F.M	(0.22, 47)
E->	Pe.S		0.195	0.195	F.M	(0.04, 0.28)
O->	Pe.S		0.065	0.065	F.M	(0.02, 0.82)
A->	Pe.S		0.006	0.006	F.M	(-0.02, 0.01)
N->	Pe.S		− 0.009	− 0.009	F.M	(-0.01, − 0.16)
C->	Ne.S		− 0.219	− 0.219	F.M	(-0.12, − 0.32)
E->	Ne.S		− 0.085	− 0.085	F.M	(-0.02, 0.21)
O->	Ne.S		− 0.112	− 0.112	F.M	(-,01, 0.22)
A->	Ne.S		0.030	0.030	F.M	(0.01, 0.07)
N->	Ne.S		0.344	0.344	F.M	(0.21, 0.42)
C->	ENG		0.247	0.247	F.M	(0.13, 0.31)
E->	ENG		0.179	0.179	F.M	(0.01, 0.24)
O->	ENG		0.067	0.067	F.M	(0.02, 0.21)
A->	ENG		0.029	0.029	F.M	(0.01, 0.34)
N->	ENG		− 0.098	− 0.098	F.M	(-0.21, − 0.02)
C->	BURN		− 0.199	− 0.199	F.M	(-0.24, − 0.02)
E->	BURN		− 0.188	− 0.188	F.M	(-0.27, − 0.01)
O->	BURN		− 0.098	− 0.098	F.M	(-0.13, 0.05)
A->	BURN		− 0.004	− 0.004	F.M	(-0.01, − 0.12)
N->	BURN		0.255	0.255	F.M	(0.36, 0.13)
C->	PFCS		0.172	0.172	F.M	(0.25, 0.02)
E->	PFCS		0.191	0.191	F.M	(0.27, 0.10)
O->	PFCS		0.052	0.052	F.M	(0.10, 0.01)
A->	PFCS		0.070	0.070	F.M	(0.14, 0.02)
N->	PFCS		− 0.120	− 0.120	F.M	(-0.15, − 0.06)
C->	EFCS		0.009	0.009	F.M	(0.015, 0.04)
E->	EFCS		0.029	0.029	F.M	(0.04, 0.11)
O->	EFCS		-,081	-,081	F.M	(-0.014, − 0.02)
A->	EFCS		,018	,018	F.M	(0.005, 0.03)
N->	EFCS		,291	,291	F.M	(0.34, 0.14)
C->	HEALTH		,067	,067	F.M	(0.10, 0.02)
E->	HEALTH		,064	,064	F.M	(0.11, 0.03)
O->	HEALTH		,067	,067	F.M	(0.10, 0.02)
A->	HEALTH		,019	,019	F.M	(0.03, 0.01)
N->	HEALTH		-,172	-,172	F.M	(-0.23, − 0.08)
SR->	Pe.S	,354		,354	D.O	(0.45, 25)
SR->	Ne.S	-,213		-,213	D.O	(-0.34, − 0.12)
SR->	ENG		,105	,105	F.M	(0.16, 0.04)
SR->	BURN		-,207	-,207	F.M	(-0.26, 0.17)
SR->	PFCS		,214	,214	F.M	(0.27, 0.16)
SR->	EFCS		-,025	-,025	F.M	(-0.04, 0.12)
SR->	HEALTH		-,034	-,034	F.M	(-0.06, 0.01)
Pos.A->	Pe.S	,381		,381	D.O	(0.46, 0.31)
Pos.A->	Ne.S		− 0.113	− 0.113	F.M	(-0.22, 0.05)
Pos.A->	ENG	,202	,187	,389	P.M	(0.42, 0.31)
Pos.A->	BURN	,00	-,232	− 0.232	F.M	(-0.31,-0.14)
Pos.A->	PFCS	,433	,046	,479	P.M	(0.53, 02)
Pos.A->	EFCS		,085	,085	F.M	(0.12, 0.05)
Pos.A->	HEALTH		,153	,153	F.M	(0.22, 07,)
Neg.A->	Ne.S	,401		,401	D.O	(0.48, 0.34)
Neg.A->	ENG		-,063	-,063	F.M	(-0.08, 0.03)
Neg.A->	BURN	,165	,142	,307	P.M	(0.09, 0.18)
Neg.A->	PFCS		-,007	-,007	F.M	(-0.07, 0.012)
Neg.A->	EFCS	0.369	,056	,425	P.M	(0.02, 0.45)
Neg.A->	HEALTH		-,257	-,257	F.M	(-0.35, − 0.18)
Pe.S->	Ne.S	− 0.269		− 0.269	D.O	(-0.32, − 0.21)
Pe.S->	ENG	,443	,046	,489	P.M	(0.52, 0.01)
Pe.S->	BURN	− 0.106	-,286	-,392	P.M	(-0.42, − 0.25)
Pe.S->	PFCS		,058	,058	F.M	(. 08, 0.03)
Pe.S->	EFCS		-,058	-,058	F.M	(-0.07, − 0.02)
Pe.S->	HEALTH		,057	,057	F.M	(0.08, 0.02)
Ne.S->	BURN	,157	,064	,221	P.M	(0.32, 0.02)
Ne.S->	PFCS	-,289	-,019	-,308	P.M	(-0.36, − 0.09)
Ne.S->	EFCS	,157	,062	,219	P.M	(0.31, 02)
Ne.S->	HEALTH	-,289	-,042	-,247	P.M	(-0.34, − 0.01)
ENG->	BURN	-,409		-,409	D.O	(-0.48, − 0.36)
ENG->	PFCS	,119		,119	D.O	(0.21, 0.15)
ENG->	EFCS		-,045	-,045	F.M	(-0.08, − 0.02)
ENG->	HEALTH		,075	,075	F.M	(0.09, 0.05)
BURN->	PFCS				N.E	
BURN->	EFCS	0.189		,189	D.O	(0.23, 0.15)
BURN->	HEALTH		− 0.106	-,106	F.M	(-0.22, -08)
PFCS ->	EFCS	,268		,268	D.O	(0.12, 0.32)
PFCS ->	HEALTH	0.419		0.419	D.O	(0.23, 51)
EFCS->	HEALTH	-,560		-,560	D.O	(-0.61, − 0.43)

Note. P.V = Predictive Variable; C = Conscientiousness; E = Extraversion; O = Openness to experience; A = Agreeableness; N = Neuroticism; SR = Self-Regulation; Pos.A = Positive Affect; Neg.A = Negative Affect; Pe.S = Positive emotions during study; Ne.S = Negative emotions during study ; ENG = Engagement; BURN = Burnout; EFCS = Emotion-focused coping strategies; PFCS = Problem-focused coping strategies; HEALTH: Health behavior; D.O = Direct Only; N.E = No effect; P.M = Partial Mediation; F.M = Full Mediation; CI = confidence interval. Bootstrapping sample size = 300

*SEM of prediction in the variables Note.* C = Conscientiousness; E = Extraversion; O = Openness to experience; A = Agreeableness; N = Neuroticism; SR = Self-Regulation; Pos.A = Positive Affect; Neg.A = Negative Affect; Pe.S = Positive emotions during study; Ne.S = Negative emotions during study; ENG = Engagement; BURN = Burnout; EFCS = Emotion-focused coping strategies; PFCS = Problem-focused coping strategies: HEALTH: Health behavior.

## Discussion

Based on the Self- vs. External-Regulation theory [27, 28], the aim of this study was to show, differentially, the regulatory, nonregulatory or dysregulatory power of the Big Five personality factors with respect to study behaviors, associated emotionality during study, motivational states, and ultimately, student health behavior.

Regarding *Hypothesis 1*, the results showed a differential, graded prediction of the Big Five personality factors affecting both self-regulation and affective states. The results from the logistic and structural regression analyses showed a clear, graded pattern from the positive predictive relationship of C to the negative predictive relationship of N. On the one hand, they showed the regulatory effect (direct and indirect) of factors C and E, the nonregulatory effect of O, and the dysregulatory effect of factors A and especially N. This evidence offers a differential categorization of the five factors in an integrated manner. On the other hand, their effects on affective tone (direct and indirect) take the same positive direction in C and E, intermediate in the case of O, and negative in A and N. There is plentiful prior evidence that has shown this relationship, though only in part, not in the integrated manner of the model presented here [29, 45–47].

Regarding *Hypothesis 2*, the evidence shows that self-regulation directly and indirectly predicts affective states in achievement emotions during study. Directionality can be positive or negative according to the influence of C and E and of positive emotionality or of A and N with negative affect. This finding agrees with prior research [29, 48–51].

Regarding *Hypothesis 3*, the results have shown clear bidirectionality. Subsequent to the prior influence of personality factors and self-regulation, achievement emotions bring about the resulting motivational states of engagement-burnout and the use of different coping strategies (problem-focused vs. emotion-focused). Positive achievement emotions during study predicted a motivational state of engagement and problem-focused coping strategies and were positive predictors of health; however, negative emotions predicted burnout and emotion-focused coping strategies and were negative predictors of health. These results are in line with prior evidence [49, 52, 53]. Finally, we unequivocally showed a double, sequenced path of emotional variables and affective motivations in a process that ultimately and differentially predicts student health [54, 55].

In conclusion, these results allow us to understand the predictive relationships involving these multiple variables in a holistic predictive model, while previous research has addressed this topic only in part [56]. We believe that these results lend empirical support to the sequence proposed by the SR vs. ER model [27]: the factors of conscientiousness and extraversion appear to be regulators of positive emotionality, engagement and health; openness to experience is considered to be nonregulating; and agreeableness and neuroticism are dysregulators of the learning process and precursors of negative emotionality and poorer student health [57]. New levels of detail—in a graded heuristic—have been added to our understanding of the relationships among the five-factor model, self-regulation, achievement emotions and health [23].

### Limitations and research prospects

A primary limitation of this study was that the analysis focused exclusively on the student. The role of the teaching context, therefore, was not considered. Previous research has reported the role of the teaching process, in interaction with student characteristics, in predicting positive or negative emotionality in students [49, 58]. However, such results do not undercut the value of the results presented here. Future research should further analyze potential personality types derived from the present categorization according to heuristic values.

### Practical implications

The relationships presented may be considered a mental map that orders the constituent factors of the Five-Factor Model on a continuum, from the most adaptive (or regulatory) and deregulatory to the most maladaptive or dysregulatory. This information is very important for carrying out preventive intervention programs for students and for designing programs for those who could benefit from training in self-regulation and positivity. Such intervention could improve how students experience the difficulties inherent in university studies [47, 59], another indicator of the need for active Psychology and Counseling Centers at universities.

## Data Availability

No datasets were generated or analysed during the current study.
